# Nutritional and Biochemical Profiling of *Leucopaxillus candidus* (Bres.) Singer Wild Mushroom

**DOI:** 10.3390/molecules21010099

**Published:** 2016-01-15

**Authors:** Vanessa Vieira, Lillian Barros, Anabela Martins, Isabel C. F. R. Ferreira

**Affiliations:** 1 Centro de Investigação de Montanha (CIMO), ESA, Instituto Politécnico de Bragança, Campus de Santa Apolónia, Apartado 1172, Bragança 5301-855, Portugal; vieira.aa.vanessa@gmail.com; 2 School of Agriculture, Polytechnic Institute of Bragança, Campus de Santa Apolónia, Ap. 1172, Bragança 5301-855, Portugal; amartins@ipb.pt

**Keywords:** *Leucopaxillus candidus*, wild mushroom, chemical composition, antioxidant activity

## Abstract

The wild mushroom *Leucopaxillus candidus* (Bres.) Singer was studied for the first time to obtain information about its chemical composition, nutritional value and bioactivity. Free sugars, fatty acids, tocopherols, organic and phenolic acids were analysed by chromatographic techniques coupled to different detectors. *L. candidus* methanolic extract was tested regarding antioxidant potential (reducing power, radical scavenging activity and lipid peroxidation inhibition). *L. candidus* was shown to be an interesting species in terms of nutritional value, with high content in proteins and carbohydrates, but low fat levels, with the prevalence of polyunsaturated fatty acids. Mannitol was the most abundant free sugar and β-tocopherol was the main tocopherol isoform. Other compounds detected were oxalic and fumaric acids, *p*-hydroxybenzoic and cinnamic acids. The methanolic extract revealed antioxidant activity and did not show hepatoxicity in porcine liver primary cells. The present study provides new information about *L. candidus*.

## 1. Introduction

*Leucopaxillus candidus* (Bres.) Singer appears between August and November in northern North America, New Mexico and Europe. The fruiting body of this mushroom is medium to large (8 to 20 cm), with convex to flat cap with the margin incurved, becoming shallowly and broadly funnel-shaped with the margin often wavy, arched, and sometimes grooved at the edge. The fruiting body exhibits whitish or pale cream, becoming slightly sordid on the disc in older specimens [1]. The stem is short, firm, full and as white as the rest of the mushroom, having tight and decurrent gills, which draw looms on the top of the cap [2]. Its edibility is controversial; there are authors claiming *L. candidus* inedibility [1], but the majority describes it as an edible source [2,3,4]. However, it can be confused with some small poisonous white Clitocybe species namely, *Clitocybe rivulosa*, *Clitocybe dealbata* and *Clitocybe cerusata* [2].

As far as we know, there are only a few studies on *L. candidus* including aspects related to its phylogeny [5,6], degradation efficiency of the explosive RDX (hexahydro-1,3,5-trinitro-1,3,5-triazine) [7], biological characterization of its mycelium [8], its major isolated metabolite-dehydromatricarianol (a polyacetylene) [9], and the possibility of gastrointestinal or resinoid syndrome caused by muscarinic ingestion [10].

Wild edible mushrooms have been included in the human diet for centuries because of their specific taste and flavour. They have been considered nutritionally healthy foods due to the high contents in carbohydrates, proteins, minerals and vitamins, and low fat levels [11]. Nowadays, they attract attention because of their bioactive compounds, beneficial effects and possible use in the prevention or treatment of diseases, being classified as functional foods and sources of nutraceuticals [12,13]. Some of the mushrooms bioactive properties are related with their antioxidant activity and antioxidant compounds [14]. In fact, antioxidants are in constant activity in living organisms, being required to be in sufficient amounts to neutralize the toxic effects of reactive oxygen species (ROS), reactive nitrogen species (RNS) and reactive sulphur species (RSS) that are produced continuously [15,16].

In the present work, *L. candidus* wild samples were submitted to a detailed study regarding chemical composition and antioxidant potential. The chemical characterization was assessed through the evaluation of nutrients (macronutrients, free sugars, fatty acids and tocopherols) and non-nutrients (organic acids and phenolic acids); the antioxidant activity of its methanolic extracts was evaluated in terms of radicals scavenging activity, reducing power and lipid peroxidation inhibition, and the confirmation of non-toxicity was carried out in a liver primary cell culture.

## 2. Results and Discussion

### 2.1. Chemical composition of L. candidus Fruiting Body

The macronutrients composition of *L. candidus* fruiting body is presented in Table 1; the moisture level was very similar to the one reported for other *Leucopaxillus* species (90 g/100 g) [17]. It showed high levels of carbohydrates (70 g/100 g dry weight, dw) and proteins (20 g/100 g dw), presenting 376 kcal/100 g dw of energetic contribution.

**Table 1:** Macronutrients, free sugars, fatty acids and tocopherols of the fruiting bodies expressed in dry weight basis (mean ± SD).

Component	*Leucopaxillus candidus*
Moisture (g/100 g)	90 ± 0.4
Fat (g/100 g)	1.8 ± 0.1
Proteins (g/100 g)	20 ± 0.2
Ash (g/100 g)	8.2 ± 0.2
Carbohydrates (g/100 g)	70 ± 1
Energy (kcal/100 g)	376 ± 1
Fructose (g/100 g)	0.35 ± 0.01
Mannitol (g/100 g)	5.6 ± 0.1
Trehalose (g/100 g)	1.1 ± 0.1
Total sugars (g/100 g)	7.1 ± 0.1
C16:0	8.0 ± 0.7
C18:0	2.7 ± 0.1
C18:1n9	37 ± 2
C18:2n6	49 ± 1
SFA (relative percentage)	13 ± 1
MUFA (relative percentage)	37 ± 2
PUFA (relative percentage)	50 ± 1
α-tocopherol (µg/100 g)	14 ± 1
β-tocopherol (µg/100 g)	33 ± 1
γ-tocopherol (µg/100 g)	3.1 ± 0.1
δ-tocopherol (µg/100 g)	7.2 ± 0.5
Total tocopherols (µg/100 g)	58 ± 1

Main fatty acids: C16:0 (Palmitic acid), C18:0 (Stearic acid), C18:1n9 (Oleic acid) and C18:2n6 (Linoleic acid); 20 more fatty acids were identified in trace amounts. SFA—Saturated fatty acids; MUFA—Monounsaturated fatty acids; PUFA—Polyunsaturated fatty acids.

Regarding the composition in free sugars, fructose, mannitol and trehalose were detected in *L. candidus* (7.1 g of total sugars/100 g) (Table 1). Mannitol was the most abundant free sugar (5.6 g/100 g), followed by trehalose (1.1 g/100 g) and fructose (0.35 g/100 g). Barros *et al.* [17] also reported mannitol and trehalose in *L. giganteus*, being trehalose the main free sugar (0.50 g/100 g fw). Mushrooms mannitol (polyol sugar) was reported to provide an inhibitory effect in angiotensin I-converting enzyme (ACE), providing hypotension of blood pressure in spontaneously hypertensive rats (SHR) [18]. Trehalose is an important component in fungal spores. Its hydrolysis is a major event during early germination and presumably serves as a source of carbon for synthesis and glucose for energy [19]. Trehalose also preserves labile proteins during drying [20]. Otherwise, the intake of dietary fructose has increased the potential of weight gain, insulin resistance syndrome, hyperinsulinemia, hypertension and hyperlipidemia in animal models [21]. The studied sample presented low content in fructose, so it has little chances to produce this kind of effects.

The fatty acids with the highest percentage in *L. candidus* (Table 1) were palmitic acid (C16:0), stearic acid (C18:0), oleic acid (C18:1n9) and linoleic acid (C18:2n6). Concerning to saturated fatty acids (SFA), palmitic acid was found in the highest percentage (8%). Oleic acid (monounsaturated fatty acid—MUFA) and linoleic acid (polyunsaturated fatty acid—PUFA) were found in higher percentages in *L. candidus* sample (37% and 49%, respectively). Consequently, this sample was richer in PUFA (50%), than in MUFA (37%) and SFA (13%). PUFA were also prevalent (47%, mainly linoleic acid—46%) over SFA (19%, mainly palmitic acid—14% and stearic acid—2%) [17]. Foods rich in ω-3 PUFA confer cardio protective effects, lowering blood pressure, preventing the development of hypertension and lowering platelet aggregation [22]. Nevertheless, mushrooms contain low amounts of fat and, therefore, the healthy effects of the various fatty acids are limited.

Concerning tocopherols, the sample presented α-, β-, γ- and δ- isoforms (Table 1). β-tocopherol was presented in the highest amount (34 µg/100 g), followed by α- (14 µg/100 g), δ- (7.2 µg/100 g) and γ- (3.1 µg/100 g) isoforms. *L. candidus* presented 57.34 µg/100 g of total tocopherols. Vitamin E is considered one of the most potent liposoluble antioxidant to retard and to prevent some age-related degenerative diseases [23]. In addition to its antioxidant nature, vitamin E has been reported to enhance immune response [24].

Regarding organic acids profile, it was possible to identify and quantify two different compounds (Table 2), namely oxalic (0.41 g/100 g) and fumaric (0.40 g/100 g) acids.

**Table 2:** Organic acids and phenolic compounds of the fruiting bodies expressed in dry weight basis (mean ± SD).

Compound	*Leucopaxillus candidus*
Oxalic acid (g/100 g)	0.41 ± 0.09
Fumaric acid (g/100 g)	0.40 ± 0.00
Total organic acids (g/100 g)	0.81 ± 0.10
*p*-Hydroxybenzoic acid (µg/100 g)	0.71 ± 0.05
*p*-Coumaric acid (µg/100 g)	0.58 ± 0.05
Total phenolic acids (µg/100 g)	1.3 ± 0.1
Cinnamic acid (µg/100 g)	0.12 ± 0.01

Concerning phenolic acids, the studied sample revealed the presence *p*-hydroxybenzoic (0.71 µg/100 g) and *p*-coumaric (0.58 µg/100 g) acids, as also the related compound cinnamic acid (0.12 µg/100 g) (Table 2). Different authors reported the organic acids profile of *L. giganteus*, another species of the same genus. Ribeiro *et al.* [25] studied the influence of different nitrogen sources in organic acids profile measured by HPLC-UV, noting citric acid as the main organic acid present (90%–96%). Barros *et al.* [26] used UFLC-PDA (ultra fast liquid chromatograph coupled to photodiode array detector), and reported malic acid as the main organic acid (6 g/100 g). Concerning to phenolic compounds profile, Barros *et al.* [27] studied the phenolic constitution of several species; however, the authors were not able to detect any compound in *L. giganteus* sample. Phenolic compounds and organic acids contribute to organoleptic properties of food matrices [28]. Furthermore, these compounds may be involved in the protection against several diseases [29]. For instance, Son *et al.* [30] reported that millimolar concentrations of oxalic acid have a strong antibrowning activity, and Ribeiro *et al.* [25] described fumaric acid as an important compound due to its antioxidant, antimicrobial and acidifying properties. However, oxalic acid is found in living organism as calcium salt, and calcium oxalate is the principal component of kidney stones and can be directly absorbed by the gut despite its insolubility [25].

### 2.2. Antioxidant Activity of the Methanolic Extracts and Confirmation of Non-Toxicity

Analysing the results of the antioxidant potential (Table 3), *L. candidus* methanolic extract revealed a strong reducing power evaluated through the *Folin-Ciocalteu* (20 mg GAE/g extract) and Ferricyanide/Prussian blue (EC_50_ = 1.04 mg/mL) assays, as also an interesting radical scavenging activity measured by DPPH assay (EC_50_ = 2.3 mg/mL). The lipid peroxidation inhibition was measured by β-carotene/linoleate (EC_50_ = 2.0 mg/mL) and TBARS (3.0 mg/mL) assays. Even though these results are much higher than the positive control trolox (which is a single molecule), they are comparable and in the same range as other mushrooms. *L. giganteus* is a species from the same genus that also has been reported as having antioxidant activity [31]. The reducing power was evaluated through the *Folin-Ciocalteu* (6.3 mg GAE/g extract) and Ferricyanide/Prussian blue (EC_50_ = 1.7 mg/mL) assays, demonstrating that *L. giganteus* had a lower reducing power than *L. candidus*. However, the same study of the authors showed best results concerning the radical scavenging activity measured by DPPH assay (1.4 mg/mL). Regarding the lipid peroxidation inhibition, the authors only used β-carotene/linoleate assay (EC_50_ = 2.0 mg/mL), reporting similar results to the present work.

**Table 3:** Antioxidant activity of the methanolic extracts (mean ± SD).

Antioxidant Activity	Assay	*Leucopaxillus candidus*
Reducing power	*Folin-ciocalteu* (mg GAE/g extract)	20 ± 1
	Ferricyanide/Prussian blue (EC_50_; mg/mL)	1.04 ± 0.01
Radical scavenging activity	DPPH scavenging activity (EC_50_; mg/mL)	2.3 ± 0.1
Lipid peroxidation inhibition	β-carotene/linoleate (EC_50_; mg/mL)	2.0 ± 0.4
	TBARS (EC_50_; mg/mL)	3.0 ± 0.3

Concerning the *Folin-Ciocalteu* assay, higher values mean higher reducing power; for the other assays, the results are presented in EC_50_ values, what means that higher values correspond to lower reducing power or antioxidant potential. EC_50_: Extract concentration corresponding to 50% of antioxidant activity or 0.5 of absorbance for the Ferricyanide/Prussian blue assay. Trolox EC_50_ values: 0.04 mg/mL (reducing power), 0.04 mg/mL (DPPH scavenging activity), 0.02 mg/mL (β-carotene bleaching inhibition) and 0.02 mg/mL (TBARS inhibition).

As the methanolic extract displayed antioxidant activity, it was important to guarantee the absence of cytotoxicity against liver cells. Mammalian hepatocytes represent an obligatory step in the evaluation of toxic compounds that lead to the production of various metabolites, which are the ultimate cause of toxicity [32,33]. Herein, porcine liver was used as an *in vitro* cytotoxicity model because it is known, in terms of cellular and physiological functioning, to be very similar to human, being cheaper and faster, and avoiding some ethical concerns related with the use of fresh human tissue (e.g., human hepatocytes) for cell lines establishment [32,33]. PLP2 culture was established in our laboratory to perform a preliminary toxicity screening of the extract, which was tested up to a maximal concentration of 3 mg/mL (maximal EC_50_ value obtained in the antioxidant activity assays). Up to the mentioned concentration, the extract did not reveal toxicity in liver primary culture PLP2 (no significant inhibition of the cells growth was observed in the presence of the extract), while the positive control ellipticine gave a GI_50_ = 2.06 ± 0.03 µg/mL.

## 3. Materials and Methods

### 3.1. Samples

*Leucopaxillus candidus* (Bres.) Singer samples were collected in Bragança (North-eastern of Portugal), in November 2012. The authentications were done in Polytechnic Institute of Bragança and a voucher specimen was deposited at the herbarium of School of Agriculture of Polytechnic Institute of Bragança (Bragança, Portugal).

Around five fruiting body samples, all in the same maturity stage, were immediately freeze-dried (FreeZone 4.5, Labconco, Kansas City, MO, USA), reduced to a fine dried powder (20 mesh), mixed to obtain a homogenate sample and stored in a desiccator, protected from light, until further analysis.

### 3.2. Standards and Reagents

Acetonitrile 99.9%, *n*-hexane 95% and ethyl acetate 99.8% were of HPLC grade from Fisher Scientific (Lisbon, Portugal). The fatty acids methyl ester (FAME) reference standard mixture 37 (standard 47885-U) was purchased from Sigma (St. Louis, MO, USA), as also other individual fatty acids, trolox (6-hydroxy-2,5,7,8-tetramethylchroman-2-carboxylic acid), sugar, tocopherol, organic acid and phenolic compound standards. Racemic tocol, 50 mg/mL, was purchased from Matreya (Pleasant Gap, PA, USA). 2,2-Diphenyl-1-picrylhydrazyl (DPPH) was obtained from Alfa Aesar (Ward Hill, MA, USA). Fetal bovine serum (FBS), Hank’s balanced salt solution, nonessential amino acids solution (2 mM), penicillin/streptomycin solution (100 U/mL and 100 µg/mL, respectively), DMEM medium was from Hyclone (Logan, Utah, USA). Acetic acid, ellipticine and sulforhodamine B (SRB) were from Sigma Chemical Co. Methanol and all other chemicals were of analytical grade and purchased from common sources. Water was treated in a Milli-Q water purification system (TGI Pure Water Systems, Topway Global, Greenville, SC, USA).

### 3.3. Chemical Composition of L. candidus Fruiting Body

*Nutritional value.* The samples were analysed for the chemical composition (moisture, proteins, fat, carbohydrates and ash) using the standard procedures [34]. The crude protein content (N × 4.38) of the samples was estimated by the macro-Kjeldahl method; the crude fat was determined by extracting a known weight of powdered sample with petroleum ether, using a Soxhlet apparatus; the ash content was determined by incineration at 600 ± 15 °C. Total carbohydrates were calculated by difference. Energy was calculated according to the following equation: Energy (kcal) = 4 × (g protein + g carbohydrate) + 9 × (g fat).

*Free sugars.* Free sugars were determined by a high performance liquid chromatograph (HPLC) system consisted of an integrated system with a pump (Smartline system 1000, Knauer, Berlin, Germany), degasser system (Smartline manager 5000, Knauer) and an auto-sampler (AS-2057, Jasco, Easton, MD, USA), coupled to a refraction index detector (RI detector Knauer Smartline 2300, Knauer) as previously described by Barros *et al.* [17]. Sugars identification was made by comparing the relative retention times of sample peaks with standards. Data were analysed using Clarity 2.4 Software (DataApex, Podohradska, Czech Republic). The chromatographic separation was achieved with a Eurospher 100-5 NH2 column (4.6 × 250 mm, 5 mm, Knauer) operating at 35 °C (7971 R column heater, Grace, Columbia, MD, USA). The mobile phase was acetonitrile/deionized water, 70:30 (*v/v*) at a flow rate of 1 mL/min. Quantification was based on the RI signal response of each standard, using the internal standard (IS, raffinose) method and by using calibration curves obtained from the commercial standards of each compound: fructose (Limit of detection—LOD: 0.05 mg/mL, Limit of quantification—LOQ: 0.18 mg/mL); mannitol (LOD: 0.07 mg/mL, LOQ: 0.22 mg/mL) and trehalose LOD: 0.07 mg/mL, LOQ: 0.24 mg/mL). The results were expressed in g per 100 g of dry weight (dw).

*Fatty acids.* Fatty acids were determined after a transesterification procedure as previously described by Barros *et al.* [17]. The analysis was carried out with a DANI model GC 1000 instrument (DANI instruments, Contone, Switzerland) equipped with a split/splitless injector, a flame ionization detector (FID at 260 °C) and a Macherey-Nagel (Düren, Germany) column (50% cyanopropyl-methyl-50% phenylmethylpolysiloxane, 30 m × 0.32 mm i.d. × 0.25 μm df). The oven temperature program was as follows: the initial temperature of the column was 50 °C, held for 2 min, then a 30 °C/min ramp to 125 °C, 5 °C/min ramp to 160 °C, 20 °C/ min ramp to 180 °C, 3 °C/min ramp to 200 °C, 20 °C/min ramp to 220 °C and held for 15 min. The carrier gas (hydrogen) flow-rate was 4.0 mL/min (0.61 bar), measured at 50 °C. Split injection (1:40) was carried out at 250 °C. Fatty acid identification was made by comparing the relative retention times of FAME peaks from samples with standards. The results were recorded and processed using Clarity 4.0.1.7 Software (DataApex) and expressed as relative percentage of each fatty acid.

*Tocopherols.* Tocopherols were determined following a procedure previously described by Heleno *et al.* [35]. Analysis was performed by HPLC (equipment described above), and a fluorescence detector (FP-2020; Jasco) programmed for excitation at 290 nm and emission at 330 nm. The chromatographic separation was achieved with a Polyamide II (250 mm × 4.6 mm i.d.) normal-phase column from YMC Waters (Lisbon, Portugal) operating at 35 °C. The mobile phase used was a mixture of *n*-hexane and ethyl acetate (70:30, *v/v*) at a flow rate of 1 mL/min. The compounds were identified by chromatographic comparisons with authentic standards: α-tocopherol (LOD: 18.06 ng/mL, LOQ: 60.20 ng/mL); β-tocopherol (LOD: 25.82 ng/mL, LOQ: 86.07 ng/mL); γ-tocopherol (LOD: 14.79 ng/mL, LOQ: 49.32 ng/mL) and δ-tocopherol (LOD: 20.09 ng/mL, LOQ: 66.95 µg/mL). Quantification was based on the fluorescence signal response of each standard, using the IS (tocol) method and by using calibration curves obtained from commercial standards of each compound. The results were expressed in μg per 100 g of dry weight (dw).

*Organic acids.* Organic acids were determined by ultra-fast liquid chromatography (UFLC, Shimadzu 20A series, Shimadzu Corporation, Kyoto, Japan) coupled with a photodiode array detector (PDA) as previously described by Barros *et al.* [26]. Separation was achieved on a SphereClone (Phenomenex, Torrance, CA, USA) reverse phase C18 column (5 μm, 250 mm × 4.6 mm i.d.) thermostatted at 35 °C. The elution was performed with sulphuric acid (3.6 mM) using a flow rate of 0.8 mL/min. Detection was carried out in a PDA, using 215 nm as the preferred wavelength. The organic acids were quantified by the comparison of the area of their peaks recorded at 215 nm with calibration curves obtained from commercial standards of each compound: oxalic acid (LOD: 12.55 µg/mL, LOQ: 41.82 µg/mL) and fumaric acid (LOD: 0.08 µg/mL, LOQ: 0.26 µg/mL). The results were expressed in g per 100 g of dry weight (dw).

*Phenolic acids and related compounds.* Phenolic acids determination was performed using the UFLC mentioned above, as previously described by Barros *et al.* [27]. A Waters Spherisorb S3 ODS-2 C18, 3 μm (4.6 mm × 150 mm) column thermostatted at 35 °C was used for chromatographic separation. The solvents used were: (A) 0.1% formic acid in water, (B) acetonitrile. The elution gradient established was isocratic 15% for 5 min, 15% B to 20% B over 5 min, 20%–25% B over 10 min, 25%–35% B over 10 min, 35%–50% for 10 min, and re-equilibration of the column, using a flow rate of 0.5 mL/min. Detection was carried out in a photodiode array detector (PDA), using 280 nm as the preferred wavelength. The phenolic compounds were quantified by comparison of the area of their peaks recorded at 280 nm with calibration curves obtained from commercial standards of each compound: *p*-hydroxybenzoic acid (LOD: 0.15 µg/mL, LOQ: 0.58 µg/mL); *p*-coumaric acid (LOD: 0.19 µg/mL, LOQ: 0.63 µg/mL) and cinnamic acid (LOD: 0.13 µg/mL, LOQ: 0.41 µg/mL). The results were expressed in µg per 100 g of dry weight (dw).

### 3.4. Bioactivity of L. candidus Methanolic Extract

*Extract preparation.* The lyophilized sample (1 g) was extracted by stirring with 40 mL of methanol for 1 h and subsequently filtered through Whatman No. 4 paper. The residue was then extracted with 20 mL of methanol for 1 h. The combined methanolic extracts were evaporated at 40 °C (rotary evaporator Büchi R-210, Flawil, Switzerland) to dryness and re-dissolved (60 mg/mL) in (a) methanol for antioxidant activity assays; (b) distillated water for the toxicity assay in porcine liver primary cells.

*Antioxidant activity assays.* Successive dilutions were made from the stock solution and submitted to the *in vitro* assays already described by Barros *et al.* [31] to evaluate the antioxidant activity of the samples. The sample concentrations (mg/mL) providing 50% of antioxidant activity or 0.5 of absorbance (EC_50_) were calculated from the graphs of antioxidant activity percentages (DPPH, β-carotene/linoleate and TBARS assays) or absorbance at 690 nm (ferricyanide/Prussian blue assay) against sample concentrations. Trolox was used as a positive control.

*Folin-Ciocalteu assay.* One of the extract solutions (5 mg/mL; 1 mL) was mixed with *Folin-Ciocalteu* reagent (5 mL, previously diluted with water 1:10, *v/v*) and sodium carbonate (75 g/L, 4 mL). The tubes were vortex mixed for 15 s and allowed to stand for 30 min at 40 °C for colour development. Absorbance was then measured at 765 nm (Analytikjena spectrophotometer; Analytikjena, Jena, Germany). Gallic acid was used to obtain the standard curve and the reduction of the *Folin-Ciocalteu* reagent by the samples was expressed as mg of gallic acid equivalents (GAE) per g of extract.

*Ferricyanide/Prussian blue assay.* The extract solutions with different concentrations (0.5 mL) were mixed with sodium phosphate buffer (200 mmol/L, pH 6.6, 0.5 mL) and potassium ferricyanide (1% *w/v*, 0.5 mL). The mixture was incubated at 50 °C for 20 min, and trichloroacetic acid (10% *w/v*, 0.5 mL) was added. The mixture (0.8 mL) was poured in the 48 wells plate, the same with deionized water (0.8 mL) and ferric chloride (0.1% *w/v*, 0.16 mL), and the absorbance was measured at 690 nm in ELX800Microplate Reader (Bio-Tek Instruments, Inc.; Winooski, VT, USA).

*DPPH radical-scavenging activity assay.* This methodology was performed using the Microplate Reader mentioned above. The reaction mixture on 96 well plate consisted of a solution by the well of the extract solutions with different concentrations (30 μL) and methanolic solution (270 μL) containing DPPH radicals (6 × 10^−5^ mol/L). The mixture was left to stand for 30 min in the dark, and the absorption was measured at 515 nm. The radical scavenging activity (RSA) was calculated as a percentage of DPPH discoloration using the equation: %RSA = [(A_DPPH_ − A_S_)/A_DPPH_] × 100, where A_S_ is the absorbance of the solution containing the sample, and A_DPPH_ is the absorbance of the DPPH solution.

*Inhibition of β-carotene bleaching or β-carotene/linoleate assay.* A solution of β-carotene was prepared by dissolving β-carotene (2 mg) in chloroform (10 mL). Two millilitres of this solution were pipetted into a round-bottom flask. The chloroform was removed at 40 °C under vacuum and linoleic acid (40 mg), Tween 80 emulsifier (400 mg), and distilled water (100 mL) were added to the flask with vigorous shaking. Aliquots (4.8 mL) of this emulsion were transferred into test tubes containing extract solutions with different concentrations (0.2 mL). The tubes were shaken and incubated at 50 °C in a water bath. As soon as the emulsion was added to each tube, the zero time absorbance was measured at 470 nm. β-Carotene bleaching inhibition was calculated using the following equation: (Absorbance after 2h of assay/initial absorbance) × 100.

*Thiobarbituric acid reactive substances (TBARS) assay.* Porcine (*Sus scrofa*) brains were obtained from a local slaughter house, dissected, and homogenized with Polytron in an ice cold Tris-HCl buffer (20 mM, pH 7.4) to produce a 1:2 *w/v* brain tissue homogenate which was centrifuged at 3000 *g* for 10 min. An aliquot (100 μL) of the supernatant was incubated with the different concentrations of the sample solutions (200 μL) in the presence of FeSO_4_ (10 mM; 100 μL) and ascorbic acid (0.1 mM; 100 μL) at 37 °C for 1h. The reaction was stopped by the addition of trichloroacetic acid (28% *w/v*, 500 μL), followed by thiobarbituric acid (TBA, 2%, *w/v*, 380 μL), and the mixture was then heated at 80 °C for 20 min. After centrifugation at 3000 g for10 min to remove the precipitated protein, the colour intensity of the malondialdehyde (MDA)-TBA complex in the supernatant was measured by its absorbance at 532 nm. The inhibition ratio (%) was calculated using the following formula: Inhibition ratio (%) = [(A − B)/A] × 100%, where A and B were the absorbance of the control and the sample solution, respectively.

### 3.5. Toxicity for Porcine Liver Cells

A cell culture was prepared from a freshly harvested porcine liver obtained from a local slaughter house. It was designed as PLP2. Briefly, the liver tissues were rinsed in Hank’s balanced salt solution containing 100 U/mL penicillin and 100 µg/mL streptomycin and divided into 1 × 1 mm^3^ explants. Some of these explants were placed in 25 cm^2^ tissue flasks in DMEM supplemented with 10% fetal bovine serum, 2 mM nonessential amino acids and 100 U/mL penicillin, 100 mg/mL streptomycin and incubated at 37 °C with a humidified atmosphere containing 5% CO_2_. The medium was changed every 2 days. Cultivation of the cells was continued with direct monitoring every 2–3 days using a phase contrast microscope. Before confluence, cells were sub-cultured and plated in 96-well plates at a density of 1.0 × 10^4^ cells/well, and cultivated in DMEM medium with 10% FBS, 100 U/mL penicillin and 100 µg /mL streptomycin [32]. Cells were treated for 48 h with the different diluted sample solutions and the same procedure described in the previous section for SRB assay. Ellipticine was used as positive control. The results were expressed in GI_50_ values (sample concentration that inhibited 50% of the net cell growth).

## 4. Conclusions

*L. candidus* proved to be a nutritionally interesting species, with high content in proteins and carbohydrates, but low fat levels and with prevalence of polyunsaturated fatty acids. Mannitol was the most abundant free sugar and β-tocopherol was the main tocopherol isoform found in this mushroom. It was possible to identify and quantify two organic acids, oxalic and fumaric acids, both with similar concentrations. *p*-Hydroxybenzoic acid was the main phenolic acid detected in the sample, but it also showed the presence of cinnamic acid. Its methanolic extract revealed antioxidant activity and did not show toxicity in porcine liver primary cells. This study provides new data concerning chemical characterization and bioactivity of *L. candidus*.
